# Management of West Nile Encephalitis: An Uncommon Complication of West Nile Virus

**DOI:** 10.7759/cureus.13183

**Published:** 2021-02-06

**Authors:** Ammar Alli, Juan Fernando Ortiz, Adam Atoot, Ali Atoot, Paul W Millhouse

**Affiliations:** 1 Internal Medicine, Hospital del Mar, Barcelona, ESP; 2 Neurology, Universidad San Francisco de Quito, Quito, ECU; 3 Neurology, California Institute of Behavioral Neurosciences & Psychology, Fairfield, USA; 4 Internal Medicine, Palisades Medical Center, North Bergen, USA; 5 Anesthesiology, Hackensack University Medical Center, Hackensack, USA; 6 General Practice, Drexel University College of Medicine, Philadelphia, USA

**Keywords:** neuroinvasive west nile virus

## Abstract

West Nile virus disease (WNVD) is a mosquito-borne disease that affects the meninges and central nervous system, causing West Nile virus (WNV) encephalitis, a debilitating, life-threatening condition, especially in the elderly. While there is a lot of research discussing different aspects of the disease, the treatment is mainly unknown. We conducted a literature review to explore the wide variety of treatment options that consolidate the knowledge about the most recent management of WNV encephalitis. We did a combined advanced search and Medical Subject Headings (MeSH) search on PubMed. Inclusion criteria included papers written in the English language and human subjects research for the past 25 years. We initially gather 110 papers, and after applying the inclusion/exclusion criteria, we end up with 30 articles for the paper's discussion. This review aims to provide clinicians with an overview of the latest approach in treating and managing hospitalized WNVD patients. It discusses case reports and the outcome of different treatment regimens done in vitro and in vivo. The study discusses all the advancements in treatment and prophylaxis and compares their effectiveness. However, more research is warranted to gain further insight to develop a single guideline for the management of this disease. This review discusses the following treatment modalities: ribavirin, interferon-alpha, intravenous immunoglobulin (IVIG), and other less-used drugs. More studies about ribavirin are needed to know if the drug is useful for WNV encephalitis. Interferon-alpha has been shown to have both protective and disease limiting properties. At the moment, there are no guidelines for the treatment of WNV encephalitis, nor is there a single Food and Drug Administration (FDA)-approved drug. For the time being, IVIG offers the best results in treating WNV encephalitis

## Introduction and background

The West Nile virus (WNV) is defined by the Centers for Disease Control and Prevention (CDC) as the leading cause of mosquito-borne disease in the continental United States (US) [1]. In 1999 to 2017, a reported 48,183 patients were infected with WNV in the US alone [2]. In 2012 several outbreaks occurred in the US, the biggest of which occurred in Dallas, Texas. In that episode, the incidence was 7.3 per 100,000 people, compared with 2.91 per 100,000 in a similar outbreak in 2006 [3].

Specialists describe two clinical presentations of the infection, West Nile Fever and WNV Encephalitis. West Nile Fever occurs in 25% of those infected, and symptoms include rapid onset of headaches, generalized fatigue, maculopapular rash, fever, and muscle pain. The fever usually lasts a few weeks before the complete resolution of symptoms. A more dangerous presentation is WNV encephalitis; this form occurs in less than 1% of those infected, with incidence showing increased correlation with age [2]. This type involves various clinical syndromes depending on the site of infection within the central nervous system (CNS); signs and symptoms can include nuchal rigidity and Kernig or Brudzinski signs in case of meningeal involvement [2,3]. Depressed or altered consciousness, lethargy, and personality changes are more common signs of cerebral involvement [2].

Patients with WNV encephalitis often suffer from muscle weakness and flaccid paralysis due to inflammation of the anterior horn cells in the spinal cord and brainstem, characterized by leukocyte infiltration and loss of neural cells [4]. The paralysis portrays the acute progression of limb weakness within 48 hours [4] and is usually asymmetric, with hyporeflexia, and without sensory impairment [2]. Around 80% of acute flaccid paralysis occurs in patients with WNV encephalitis. In these cases, the disease persists, typically weeks to months, and long-term cognitive and functional complications may result [2].

Diagnosis of WNV encephalitis is based on clinical findings and by detecting immunoglobulin M (IgM) antibodies in serum or cerebrospinal fluid (CSF) [2,5]. Routine clinical laboratory studies are frequently nonspecific, and magnetic resonance imaging (MRI) is often normal unless brain involvement and flaccid paralysis are present [6]. Table 1 summarizes the main clinical features, pathophysiology, MRI findings and diagnosis of WNV encephalitis [1,5,7].

**Table 1:** Epidemiology, Clinical Features, Pathophisiology, MRI findings, and Diagnosis of West Nile Encephalitis CSF: Cerebrospinal fluid, US: United States, IgM: Immunoglobulin M, WNV: West Nile virus

Epidemiology, Clinical Features, Pathophisiology, MRI findings, and Diagnosis of West Nile Encephalitis	
Epidemiology [1]	Endemic in the US. Only 20% of patients develop West Nile Fever. Neuroinvasive disease is seen in less <1%, with a mortality rate of 10%. Old age increases the risk of developing WNV encephalitis
Clinical Features [7]	West Nile Fever: headache, fever, myalgia, generalized weakness, skin rash, lymphadenopathy, vomiting, and diarrhea. WNV encephalitis: West Nile meningitis and encephalitis: fever, signs of meningeal irritation like headache, nuchal rigidity, Kernig’s and Brudzinski’s signs on physical exam, altered level of consciousness, disorientation, and focal neurological signs and symptoms (eg, dysarthria, seizures, tremor) Acute flaccid paralysis: mild flu-like symptoms including headache, fever, malaise, gastrointestinal symptoms precede the onset of paralysis in which the most prominent feature is the limb weakness. Sensory examination and deep tendon reflexes are usually preserved.
Pathophisiology [7]	WNV is transmitted through infected mosquito bites. In the skin, WNV replicates in the dendritic cells. The cells then spread the infection to the regional lymph nodes and from there into the bloodstream. The mechanism of neural transmission is still unclear. However, theories propose a retrograde axonal transport of WNV in the peripheral nervous system. CNS expression of CCR5 and its ligand CCL5 is upregulated by WNV. The secretion of chemokine CXCL10 stimulates the influx of immune cells that express CCR5, mostly CD4+, CD8+, and macrophages, into the CNS by WNV infected neurons. The infiltration of lymphocytes in the CNS is the leading cause of neurological damage, which results in the clinical manifestation of WNV encephalitis.
MRI Findings [7]	Normal in most patients. Lesions were noticed on T2-weighted images in the basal ganglia (substantia nigra) pons, and thalamus. In the spinal cord, lesions consistent with anterior horn damage were also reported in patients who developed paralysis.
Diagnosis [5]	Clinical Criteria: Having the classical clinical features of encephalitis, acute flaccid paralysis, meningitis, or other central or peripheral nervous signs. These clinical signs need to be present in the absence of a better explanation. Having the following related symptoms help yield the diagnosis: myalgia, vomiting, paresis, arthralgia and or nuchal rigidity. Laboratory criteria: Isolation of the virus in the blood, CSF, or other body fluid. In the serum the IgM is detected while in the CSF is addition to the IgM, pleocytosis and negative results for arbovirus.

There are many publications about the clinical features and diagnosis of West Nile fever and encephalitis. However, The management of WNV encephalitis remains mainly unknown. Most clinical trials involve another flavivirus such as Japanese or San Louis encephalitis [8]. There is relatively little information related to West Nile fever and the associated encephalitis. Our objective was to investigate the management and prognosis of WNV encephalitis, consolidate the knowledge of this neurological infection, and establish possible management when a patient presents with this condition.

## Review

Methods

A combination of PubMed Medical Subject Headings (MeSH) search was conducted using headings like “West Nile Virus” and “West Nile encephalitis”, and Advanced PubMed search using combinations like (ribavirin[Title/Abstract]) AND (west nile virus[Title/Abstract]),  (west nile virus [Title/Abstract]) AND (interferon[Title/Abstract]) , (west nile virus[Title/Abstract]) AND (IVIG[Title/Abstract]), (west nile virus[Title/Abstract]) AND (vaccine[Title/Abstract]), and (west nile virus[Title/Abstract]) AND (supportive care[Title/Abstract]). This search strategy was intended to cover all possible articles on WNV encephalitis and its management. Each of the studies that appeared in the search results were later analysed through the study’s inclusion and exclusion criteria.

Table 2 documents the initial results taken with our methodology.

**Table 2:** Methodology of the study

Search results	Number of publications
(ribavirin[Title/Abstract]) AND (west nile virus[Title/Abstract])	3
(west nile virus [Title/Abstract]) AND (interferon[Title/Abstract])	20
(west nile virus[Title/Abstract]) AND (IVIG[Title/Abstract])	2
(west nile virus[Title/Abstract]) AND (vaccine[Title/Abstract])	67
(west nile virus[Title/Abstract]) AND (supportive care[Title/Abstract])	8

Results

Following the recollection of initial data, the results were scanned and filtered by human subjects and papers published in English language in the last 25 years. Then the abstracts were reviewed and assessed according to the study’s inclusion and exclusion criteria. Only articles addressing WNV encephalitis disease and treatment were included. 

The final search (both MeSH and advanced PubMed search) yielded a database of 110 articles. These articles were narrowed down to 42 after inclusion/exclusion criteria. We excluded an additional 12 papers after removing duplicate papers and studies that did not meet the outcomes of our objectives. Table 3 summarizes the approach followed in producing this article from initial search results to the included studies.

**Table 3:** Results of the study

Total Records	110
Inclusion criteria: Full papers published in English in the last 25 years Free full text	74
Exclusion criteria:Study type that were Systematic Reviews, Literature Reviews, or Meta Analysis	42
Exclusion of duplicates and studies that did not meet the outcomes of our objectives	30

Discussion

The Flavivirus genus is a group of highly virulent, widespread, single-stranded ribonucleic acid (RNA) positive viruses and is well known to cause many dangerous infectious diseases. The group includes but is not limited to West Nile virus (WNV), dengue virus (DENV), Zika virus (ZIKV), and yellow fever virus (YFV). The most devastating pathogenic property of these viruses is that they have animal reservoirs that serve as a constant source of outbreaks [9]. In other words, epidemics caused by these viruses will keep emerging whenever the right conditions are met. Thus, finding treatment for these different viruses is crucial to avoid health and economic threats to local and international communities. To date, there is no Food and Drug Administration (FDA)-approved treatment for infection with WNV. However, there are some drugs with promising results. We will examine the efficacy of interferon alpha 2b (INF α2b), ribavirin, intravenous immunoglobulin (IVIG), and other less-used medications to treat WNV encephalitis. Then we will discuss the supportive treatment and prophylaxis of the virus with vaccines.

Interferon 

In vitro studies of INF α2b have shown efficacy in decreasing the activity of WNV [8,10]. INF α2b works by inhibiting the virus's replication by blocking viral polypeptides [11]. In studies in vitro, INF α2b inhibited viral cytotoxicity at low doses when applied before introducing the virus into cells; it also demonstrated having therapeutic properties when it was administered to cells that had already been infected [8].

In a case report with two patients who developed WNV encephalitis, there was benefit from the use of INF α2b on days four and six. On day six, the patient who received treatment had more sequels than those received on day four [4]. In a report by Lewis et al., a patient was successfully treated with INF α2b, 14 days after the hospital's admission [11]. It is important to note that studies done in mice encountered that giving INF α2b before two days had better outcomes than mice who received after [11]. In a case series of three of WNV encephalitis, all recovered after receiving INF α2b on days five, eight, and nine after the onset of symptoms. Two patients had WNV encephalitis and cerebellar signs. At the same time, the other patient had WNV encephalitis and neuromuscular weakness [12]. The first case reported of WNV encephalitis and myelitis in Korea reported a 58-year man who recovered three months after receiving interferon-alpha at day 14 of the onset of symptoms [13].

In contrast to these three previous reports, a case of a 76-year-old man receiving interferon-alpha on day 17 of treatment, but he still died [13]. These findings suggest that INF α2b is more beneficial when given early. However, INF α2b benefit could happen even 14 days after the onset of symptoms, but with less success rate.

INF α2b was effective in vitro against some flaviviruses [14]. For this reason, interferon was presumptuously administered during the 2000 Israeli outbreak to help treat WNV infection but no benefit of any kind was recorded. In a 2002 US outbreak, a randomised clinical trial, INF α2b vs. placebo-centered trial in patients with Japanese encephalitis, failed [14]. These results lead to the need to question the effectiveness of these drugs in vivo.

Ribavarin

Ribavirin is a guanosine analog that inhibits inosine-5′-monophosphate (IMP) dehydrogenase, limiting viral replication [15]. Ribavirin has shown in vitro efficacy over a wide range of RNA and DNA viruses, including Flaviviridae, which encouraged its use against the WNV. An in vitro study focused on the effects of ribavirin in cultured human oligodendroglial cells. The in vitro study demonstrated reduction in the replication rate of the virus [15].

Regarding human studies, a four-year-old boy with Hodgkin's lymphoma was admitted to the hospital after his third session of chemotherapy with high fever and no evidence of meningeal irritation; the physical exam was unremarkable. His condition deteriorated three days later as he became lethargic, disoriented, and experienced repeated episodes of generalized seizures. Physical examination showed nuchal rigidity. His mental status deteriorated, becoming somnolent. Serum IgM for WNV came back positive. On the eighth day after admission, he received ribavirin for 14 days. At the beginning of the third week, a slow improvement was noted. With the help of supportive care, he was dismissed 28 days after admission [16].

Two patients receiving ribavirin and INF α2b for the treatment of hepatitis C developed west virus fever. While it is true that the patients did not develop encephalitis, this finding questions the limited utility of both drugs [17].

Immunoglobulins 

There are two types of IVIG for the use of WNV encephalitis. First, the plasma-gathered immunoglobulins donated by survivors and the second, the pharmaceutical West Nile immunoglobulins (WNIG) [18]. The IVIGs interaction between the Fc portion of immunoglobulin IgG (IgG-Fc) and Fc Receptors (FcR) has an anti-inflammatory effect, mainly due to sialic acid's residues on the IgG Fc terminal. This part of the antibodies has demonstrated a protective role against autoimmune conditions such as rheumatoid arthritis and immune thrombocytopenia in laboratory mice [16].

In mouse studies, convalescent attained immunoglobulins achieved a protective rate of 44%. At the same time, the WNIG protection levels neared 94% [18]. In another mouse study, administration of WNIG up to four days post-infection demonstrated survival rates of 63% compared to 25% in the control group. In comparison, no significant difference in survival was observed when WNIG was administered after five days or more of infection, as the neurological injury after this period became irreversible [16].

A patient with a liver transplant developed a fever return to the hospital 15 days after the operation. On day four of hospitalization, he received 0.4 g/kg of IVIG on suspicion of WNV encephalitis. The improvement began on day five of treatment. He was discharged on day 12 of his hospital stay [19]. A case series of three patients' recipients of organ transplants developed WNV encephalitis and were treated with WNV IVIG. Two of the three patients completely recovered while the third died [20]. Finally, A 55-year-old man with leukemia was lethargic. He had positive IgM consistent with WNV encephalitis. He received 0.5 g/kg WNV immunoglobulin for six days. However, his condition continued to deteriorate until he died. It's unclear to what degree his underlying chronic lymphocytic leukemia and associated hypoimmunoglobulinemia contributed to the acquisition of infection in the first place [21].

In general, most studies support the administration of IVIG to patients with WNV encephalitis. Data indicate that IVIG should be administered with a dose of 0.4g/kg for five days. The average hospital stay was 15 days but may vary according to the patient's underlying conditions [21].

Other Therapies (Gamma Interferon and ETAR)

It is believed that gamma interferon has a protective role against replication and spread of WNV. Research has shown that mice with low levels of gamma interferon or gamma interferon receptors were more susceptible to severe WNND and had lower survival rates than their counterparts with normal levels. Higher levels of WNV RNA were isolated from serum, brain, and spleen tissues of interferon-deficient mice. Interferon-gamma seems to slow down the infection, allowing more time for the adaptive immune response to develop [22].

An analog of 21 nucleosides similar to ribavirin,1-β-D-ribofuranosyl-3-ethynyl-(1,2,4) triazole (ETAR) has shown more efficacy comparable cytotoxicity to that of ribavirin in vitro. The medicine covers a wide range of flaviviruses. It requires a lower effective dose than ribavirin when used in vitro, but further research is needed on ETAR to test its efficacy and cytotoxicity compared to ribavirin in vivo [10].

Vaccines

Prophylaxis measures against WNV have been studied since 1990. One of these measures was the development of vaccines both for human and veterinary use. Equine vaccines have successfully protected the virus, and several have been authorized for veterinary purposes [23]. No vaccine is yet available to humans. However, many promising vaccines were under development. Studies have shown that protein E is an essential target for almost all successful vaccines [23]. Protein E is an envelope protein responsible for viral entry into host cells. Therefore, it's a critical target for neutralizing antibodies [23].

All of these vaccines underwent phase I of development and were then suspended for various reasons. One of the main reasons was the unknown time interval between outbreaks in a given region. Choosing phase III's target population and follow-up with them could be difficult and cost-ineffective [14,24]. There are other regions where other flaviviruses co-circulate. In these regions, it would be the production of antibodies against other flaviviruses, which may protect against WNV. The mechanism involved is through cross-reaction, making administering the vaccine in such areas unnecessary [25]. None of the vaccines resulted in any side effects or safety issues that could proceed with clinical trials to halt. The three most promising vaccines are displayed in Table 4 [26-28].

**Table 4:** Vaccines for West Nile Virus WNV: West Nile virus

Vaccine	Properties	Efficacy
ChimeriVax-WN02 [26]	Recombinant yellow fever vaccine using fragments of protein M and E of WNV	After one dose More than 90% neutralizing antibodies in older and younger patients
rWN/DEN4Δ30 [27]	Recombinant attenuated Dengue virus expressing fragments of protein M and E of WNV	Two doses Neutralizing antibodies 89%
VRC WNV [28]	DNA plasmid expressing protein M and E of WNV	Three doses More than 90% neutralizing antibodies in young and old age groups

Supportive Care

To date, supportive care continues to be the main treatment for WNV encephalitis. Pain management is crucial in these patients since the most common neurological symptom is headaches 69.7% after hospital admission [29]. Support care includes treating infections with antibiotics. Convulsions and respiratory failure may necessitate intensive care [30]. Administration of mannitol and steroids is also practiced in many hospitals to better control symptoms [29]. Patients with WNV encephalitis usually suffer from long-term neurological impairment that starts to improve six to eight weeks after the infection, and improvement usually reaches a limit after 12 weeks. Therefore, patient follow-up is advised with a two to six-month interval [29]. One factor that has played a role in improving treatment outcomes and helps protect against complications is the time interval between the onset of neurological symptoms and admission to the hospital. The longer the gap, the more severe the neurological state will be [29].

## Conclusions

West Nile virus is an endemic infection in the United States that continues to be a threat to different communities across the country with no cure to date. Although its neuroinvasive form is relatively rare, it causes serious morbidity and mortality risk to those affected. The length of hospital stay and leave of absence are only two examples of the financial costs that weigh on families and health care systems alike. More studies about ribavirin are needed to know if the drug is useful for WNV encephalitis. Interferon-alpha has been shown to have both protective and disease limiting properties. The drug seems to work better when given nine to 10 days after the onset of symptoms. At the moment, there are no guidelines that determine the course of treatment, nor is there a single FDA-approved drug. For the time being, IVIG offers the best results in treating WNV neuroinvasive disease. As the annual cases continue to increase worldwide, finding a cure is becoming more crucial with time.
